# Comprehensive bactericidal activity of an ethanol-based hand gel in 15 seconds

**DOI:** 10.1186/1476-0711-7-2

**Published:** 2008-01-22

**Authors:** Günter Kampf, Angela Hollingsworth

**Affiliations:** 1 Bode Chemie GmbH & Co. KG, Scientific Affairs, Melanchthonstr. 27, 22525 Hamburg, Germany; 2 Institute for Hygiene and Environmental Medicine, Ernst Moritz Arndt University, Walther-Rathenau-Str. 49a, 17489 Greifswald, Germany; 3 MICROBIOTEST Inc., 105B Carpenter Drive, Sterling, Virginia, USA

## Abstract

**Background:**

Some studies indicate that the commonly recommended 30 s application time for the post contamination treatment of hands may not be necessary as the same effect may be achieved with some formulations in a shorter application time such as 15 s.

**Method:**

We evaluated the bactericidal activity of an ethanol-based hand gel (Sterillium^® ^Comfort Gel) within 15 s in a time-kill-test against 11 Gram-positive, 16 Gram-negative bacteria and 11 emerging bacterial pathogens. Each strain was evaluated in quadruplicate.

**Results:**

The hand gel (85% ethanol, w/w) was found to reduce all 11 Gram-positive and all 16 Gram-negative bacteria by more than 5 log_10 _steps within 15 s, not only against the ATCC test strains but also against corresponding clinical isolates. In addition, a log_10 _reduction > 5 was observed against all tested emerging bacterial pathogens.

**Conclusion:**

The ethanol-based hand gel was found to have a broad spectrum of bactericidal activity in only 15 s which includes the most common species causing nosocomial infections and the relevant emerging pathogens. Future research will hopefully help to find out if a shorter application time for the post contamination treatment of hands provides more benefits or more risks.

## Introduction

The use of alcohol-based hand antiseptics has become a standard worldwide to prevent the transmission of nosocomial pathogens by the hands of the healthcare workers [1]. This development was enforced after the publication of the CDC guideline on hand hygiene [2] and will be even further enforced after publication of the upcoming WHO guideline on hand hygiene [3]. Most hand rubs are tested and recommended with an application time of 30 s [4-6]. Recent data, however, indicate that with some hand antiseptics the same efficacy may also be found in shorter application times such as 15 s when the hand antiseptic is applied correctly [7].

It has been recommended that a hand hygiene agent should have at least activity against bacteria, yeasts, and enveloped viruses [1]. A Propanol-based hand rub has been described before to have a broad spectrum of bactericidal activity within 30 s [8]. Some alcohol-based hand rubs have been described to easily inactivate clinically relevant enveloped viruses in only 15 s, even when the antiseptics were challenged with various types of organic load [9]. There may be the potential to reduce the recommended application time for the post contamination treatment of hands with well formulated hand antiseptics. In that respect it is essential to know if the clinically relevant pathogens are killed under the stringent test conditions of 15 s. We have therefore studied the bactericidal activity of an ethanol-based hand gel with an exposure time of only 15 s.

## Method

### Hand rub

A hand gel based on 85% (w/w) ethanol was used for all experiments (Sterillium Comfort Gel, Bode Chemie GmbH & Co. KG, Hamburg, Germany). It was used because many other alcohol-based hand gels with a lower concentration of ethanol or propanol such as 70% or less were described to be significantly less effective than the European reference treatment and are not recommended for use in hospitals due to the limited efficacy [4].

### Test bacteria – ATCC strains

The following ATCC strains were used: *Acinetobacter baumannii *19606, *Acinetobacter lwoffi *15309, *Bacteroides fragilis *25285, *Burkholderia cepacia *25416, *Clostridium difficile *9689, *Enterobacter aerogenes *13048, *Enterobacter cloacae *13047, *Enterococcus faecalis *29212, *Enterococcus faecium *19434, *Escherichia coli *11229 and 25922, *Haemophilus influenzae *19418, *Klebsiella pneumoniae *11296, *Klebsiella oxytoca *43165, *Listeria monocytogenes *7644, *Micrococcus luteus *7468, *Proteus mirabilis *7002, *Pseudomonas aeruginosa *15442 and 27853, *Salmonella enteritidis *13076, *Salmonella typhimurium *13311, *Serratia marcescens *14756, *Shigella sonnei *11060, *Staphylococcus aureus *6538 and 29213, Methicillin-resistant *Staphylococcus aureus *33591, *Staphylococcus epidermidis *12228, *Staphylococcus haemolyticus *29970, *Staphylococcus hominis *27844, *Staphylococcus saprophyticus *15305, *Streptococcus pneumoniae *6304 and *Streptococcus pyogenes *19615.

### Test bacteria – clinical isolates

For each of the bacterial species from the ATCC strains a clinical isolate was tested as well with the exception of *A. baumannii *and *A. lwoffi *where clinical isolates were not available. *Acinetobacter calcoaceticus *var. lwoffi was chosen as the clinical corresponding isolate for both species. In addition the following emerging pathogens were included:

⇒ multidrug-resistant *Acinetobacter baumannii*, resistant to Ampicillin, Cefazolin, Ceftazidime, Ceftriaxone, Gentamicin, Tobramycin, Ciprofloxacin, Levofloxacin and Trimethoprim-sulfamethoxazole,

⇒ Vancomycin-resistant *Enterococcus faecalis *(VRE), resistant to vancomycin, Gentamicin (in conjunction with Quinolone, Vancomycin or Penicillin) and Streptomycin (in conjunction with Quinolone, Vancomycin or Penicillin)

⇒ Vancomycin-resistant *Enterococcus faecalis *(VRE), resistant to Vancomycin,

⇒ Vancomycin-resistant *Enterococcus faecium *(VRE), resistant to Vancomycin,

⇒ *Escherichia coli *0157:H7,

⇒ multidrug-resistant *Escherichia coli*, resistant to Ampicillin, Cefazolin, Ceftazidime, Ceftriaxone, Gentamicin, Tobramycin, Ciprofloxacin, Levofloxacin, Bactrim and Piperacillin/Tazobactam,

⇒ multidrug-resistant *Klebsiella pneumoniae*, resistant to Ampicillin, Cefazolin, Ceftazidime, Ceftriaxone, Gentamicin, Tobramycin, Ciprofloxacin, Levofloxacin, Trimethoprim-sulfamethoxazole and Piperacillin/Tazobactam,

⇒ multidrug-resistant *Pseudomonas aeruginosa*, resistant to Ampicillin, Cefazolin, Ceftazidime, Ceftriaxone, Trimethoprim-sulfamethoxazole and Piperacillin/Tazobactam,

⇒ methicillin-resistant *Staphyloccocus aureus*, resistano to oxacillin,

⇒ Vancomycin-intermediate-resistant *Staphylococcus aureus *(VISA), intermediate resistant against Vancomycin and other glycopeptides (reduced susceptibility),

⇒ Vancomycin-intermediate-resistant *Staphylococcus epidermidis *(VISE), intermediate resistant against Vancomycin and other glycopeptides (reduced susceptibility), and

⇒ Penicillin-resistant *Streptococcus pneumoniae *(PRSP), resistant to Oxacillin.

All isolates were received from various hospital sources. The susceptibility of the isolates was tested using the Kirby-Bauer disk diffusion methods, CSLI (formerly NCCLS) interpretive standards were applied [10].

### Test procedure

Inocula were prepared by transfer of bacteria from stock cultures into the appropriate broth media which were incubated for 18 – 24 h at 37 ± 2°C. *Haemophilus influenzae *and *Streptococcus pyogenes *were incubated in the presence of 5% carbon dioxide, *Bacteriodes fragilis *and *Clostridium difficile *were incubated under anaerobic conditions. BBL GasPak^® ^Jar Systems were used to produce the anaerobic conditions. The GasPak system generates an anaerobic environment by means of a carbon dioxide and hydrogen generator, water, and a palladium catalyst. For *Clostridium difficile *only the vegetative cell form was investigated because it is known for more than 100 years that ethanol has no or little activity against bacterial spores [11-15] which is also supported by clinical data [16,17]. The bacterial test suspension served as the control (no exposure) and the pre-value.

Ninety-nine ml of the hand antiseptic were dispensed into four sterile flasks containing stir bars as described before [8]. Flasks were allowed to equilibrate to ambient room temperature for at least 10 min. One ml of bacterial test suspension was added to each flask. After the exposure time of 15 s an aliquot of 1 ml was transferred into tubes containing 9 ml Dey/Engley (D/E) neutralizing broth. Serial ten-fold dilutions were performed in phosphate buffered dilution water. Duplicate aliquots from selected dilutions were plated using the appropriate agar and plating technique. Nutrient agar pour plates were used for *Acinetobacter baumannii*, *Burkholderia cepacia, Enterobacter aerogenes*, *Enterobacter cloacae*, *Escherichia coli *(with the exception of *Escherichia coli*, ATCC 25922), *Klebsiella pneumoniae*, *Klebsiella oxytoca*, *Proteus mirabilis*, *Pseudomonas aeruginosa*, *Salmonella enteritidis*, *Salmonella typhimurium*, *Serratia marcescens*, *Shigella sonnei*, *Staphylococcus aureus*, *Staphylococcus epidermidis*, *Staphyloccocus haemolyticus*, *Staphylococcus hominis*, and *Staphylococcus saprophyticus*. Tryptic soy agar (TSA) pour plates were used for *Escherichia coli*, ATCC 25922 and *Micrococcus luteus*. TSA containing 5% defibrinated sheep's blood plates using both spread and filter plating technique were used for *Enterococcus faecalis*, *Streptococcus pneumoniae *and *Streptococcus pyogenes*. TSA containing 5% defibrinated rabbit's blood plates using both spread and filter plating technique were used for *Listeria monocytogenes*, ATCC 7644. For membrane filtration, the filter was applied aseptically with a forceps onto the stainless steel support. The disposable funnel was snapped into place on the filtration system. The membrane filter was wet with sterile diluent (0.85% saline). The dilution tube was thoroughly mixed prior to sampling. The sample from the dilution tube was placed onto the filter surface, diluent was added and vacuum applied, using a vacuum pump and collection flask set up. The filter was removed aseptically from the filter holder with flamed forceps and place onto the surface of an agar plate [18]. Brain heart infusion agar pour plates were used for *Acinetobacter lwoffi*, *Enterococcus faecium *and *Listeria monocytogenes *(clinical isolate strain). Reinforced clostridial medium pour plates were used for *Bacteroides fragilis *and *Clostridium difficile*. Plates were incubated for the appropriate time and temperature as follows: *Haemophilus influenzae *for 36 to 50 h at 37 ± 2°C in the presence of 5% carbon dioxide, *Bacteriodes fragilis *and *Clostridium difficile *for 60 to 74 h at 37 ± 2°C under anaerobic conditions, and all remaining bacteria for 36 to 50 h at 37 ± 2°C in ambient air. After incubation colonies were counted and the number of colony-forming units (CFU) per ml calculated and transferred into a log_10 _value.

### Statistical analysis

Each experiment was carried out in quadruplicate. The log_10 _reduction factor (RF) was calculated as the difference of the number of CFU per ml before and after exposure to the hand rub using the following formula:

RF = log_10 _cfu (control) - log_10 _cfu (hand gel)

The lowest RF of the four experiments with each strain is presented.

## Results

The ethanol-based hand gel reduced all 11 Gram-positive and all 16 Gram-negative bacteria within 15 s by more than 5 log_10_-steps (Tables 1 and 2), not only against the ATCC test strains but also against corresponding clinical isolates. In addition, a RF > 5 was observed against all tested emerging bacterial pathogens (Table 3). The lowest RF was always beyond the limit of detection.

**Table 1:** Activity of Sterillium^® ^Comfort Gel against 11 Gram-positive ATCC strains and clinical isolates (15 s exposure time).

Bacterial species	Lowest RF (ATCC strain)	Lowest RF (clinical isolate)
*Enterococcus faecalis*	7.06	7.34
*Enterococcus faecium*	7.29	6.90
*Listeria monocytogenes*	6.34	6.23
*Micrococcus luteus*	5.48	5.38
*Staphylococcus aureus *including MRSA	6.29	6.58
Staphylococcus epidermidis	5.82	5.60
*Staphylococcus haemolyticus*	5.34	6.16
*Staphylococcus hominis*	5.38	5.41
*Staphylococcus saprophyticus*	6.60	5.41
*Streptococcus pneumoniae*	5.34	5.60
*Streptococcus pyogenes*	6.14	5.48

Experiments were done according to the US tentative final monograph; presentation of the lowest reduction factor (RF) of four replicate experiments for each test strain.

**Table 2:** Activity of Sterillium^® ^Comfort Gel against 16 Gram-negative ATCC strains and clinical isolates (15 s exposure time).

Bacterial species	Lowest RF (ATCC strain)	Lowest RF (clinical isolate)
*Acinetobacter baumannii*	6.60	5.34*
*Acinetobacter lwoffi*	6.86	5.34*
*Bacteriodes fragilis*	6.72	6.58
*Burkholderia cepacia*	6.48	5.48
*Enterobacter aerogenes*	6.83	5.91
*Enterobacter cloacae*	6.45	6.75
*Escherichia coli*	6.64	6.72
*Haemophilus influenzae*	5.86	5.34
*Klebsiella pneumoniae*	6.53	6.62
*Klebsiella oxytoca*	6.73	6.62
*Proteus mirabilis*	6.78	6.68
*Pseudomonas aeruginosa*	6.56	6.73
*Salmonella enteritidis*	6.79	6.75
*Salmonella typhimurium*	6.72	6.68
*Serratia marcescens*	6.87	5.62
*Shigella sonnei*	6.28	6.41

Experiments were done according to the US tentative final monograph; presentation of the lowest reduction factor (RF) of four replicate experiments for each test strain; *a clinical isolate was obtained as *Acinetobacter calcoaceticus *var lwoffi.

**Table 3:** Activity of Sterillium^® ^Comfort Gel against 11 emerging bacterial pathogens (15 s exposure time).

Bacterial species	Lowest RF
*Acinetobacter baumannii *MDR	6.45
*Clostridium difficile *(vegetative cell form)	5.34
*Enterococcus faecalis *VRE	7.15
*Enterococcus faecium *VRE	6.70
*Escherichia coli *0157:H7	6.48
*Escherichia coli *MDR	6.58
*Klebsiella pneumoniae *MDR	6.45
*Pseudomonas aeruginosa *MDR	7.22
*Staphylococcus aureus *VISA	6.38
*Staphylococcus epidermidis *VISE	6.70
*Streptococcus pneumoniae *PRSP	5.38

Experiments were performed according to the US tentative final monograph; presentation of the lowest reduction factor (RF) of four replicate experiments for each test strain.

## Discussion

It has been suggested that a hand rub should have at least activity against bacteria, yeasts and enveloped viruses [1]. In our study only the bactericidal activity of a hand gel based on 85% (w/w) ethanol was looked at. We were able to show for the first time that the tested gel kills the most relevant nosocomial bacterial pathogens in only 15 s. Ethanol is known to have a strong bactericidal activity with log_10 _reductions > 5 which has been demonstrated against some of the most common nosocomial pathogens such as *Staphylococcus aureus *and *Pseudomonas aeruginosa *[7,19-22] and which includes activity against various mycobacteria [23-25]. Nevertheless, this type of comprehensive bactericidal activity as described in our study has so far only been demonstrated with a propanol-based hand rub and an exposure time of 30 s [8].

Suspension tests, however, are not the critical part in the assessment of the efficacy assessment of alcohol-based hand antiseptics [26]. In a study with tests under practical conditions for hygienic hand disinfection (EN 1500) significant differences were observed between various alcohol-based preparations with quite poor results for those containing only up to 70% alcohol [4]. Recent data confirm that the efficacy of some alcohol-based hand antiseptics may be fairly low under practical test conditions (TFM test) especially when the overall concentration of alcohols is below 70% in the gel [27]. Suspension tests, however, can be considered to be less sensitive but are nevertheless very important to determine a general spectrum of antimicrobial activity.

We found that the gel kills the vegetative cell form of *Clostridium difficile *within 15 s which has been described before with a propanol-based hand rub in 30 s [8]. But with the emergence of *Clostridium difficile *in Europe and North America [28,29] it is important to understand that alcohols like ethanol or propanol have little or no activity against bacterial spores [11-15] which were not investigated in the present study. Therefore, it would be wrong to conclude that the application of alcohol-based hand rubs is sufficient when contamination of hands with *Clostridium difficile *is expected because both the vegetative cell form and the bacterial spore must be expected on hands next to each other. Promoting the use of alcohol-based hand rubs has been very effective to reduce the number of nosocomial infections by various bacterial species such as MRSA or VRE [16] but did not reduce or increase the number of *Clostridium difficile *cases in hospitals [16,17]. *Clostridium difficile*, however, is never the single nosocomial pathogen on healthcare workers hands. Boyce et al. showed that fecal samples of patients with *Clostridium difficile *contain also in 9.8% MRSA [30]. That is why the best hand hygiene procedure seems to be to initially perform a hand disinfection in order to kill all clinically relevant bacteria including the vegetative cell form of *Clostridium difficile*. Immediately thereafter a thorough 10 s hand wash with plain soap should be done in order to reduce the number of spores on hands as much as possible [15]. A longer hand wash or use of an antimicrobial soap do not yield a better reduction of bacterial spores on hands [15] but can substantially damage the skin [31]. Performing only a hand wash without a hand disinfection does not take into account the vast majority of nosocomial bacterial pathogens which will still be there when *Clostridium difficile *outbreaks occur.

The main argument for a shorter application time in hand disinfection without any reduction of efficacy is certainly that it is easier to comply with [32]. The overall required time will be shorter [33] which can have a positive effect on the unknowingly attitude of healthcare workers towards performing a hand disinfection procedure. A high compliance rate in hand hygiene is a key issue for a successful prevention of nosocomial infections [34]. A shorter application time such as 15 s may well make it easier for the healthcare worker to comply with the recommended standard. At the same time a shorter application time such as 15 s may result in an incomplete coverage of the hand with the antiseptic agent as suggested recently [35]. But at the same time we have to realize that there is also no evidence to show that a 30 s application time generally ensures a complete coverage of hands. If a shorter application time results in a higher compliance rate in hand hygiene, it is likely to significantly reduce the rate of nosocomial infections which will be welcomed by all professionals in infection control. On the other side it is unknown if a twinkle-toed technique with some untreated skin areas foils the expectable effect on the nosocomial infection rate at all. May be the leaks are mostly at parts of the skin which are not relevant for the transmission of pathogens. But may be the leaks are mostly at skin sites which abolish the effect of the hand disinfection procedure all together. As long as this issue can not be solved scientifically, it remains unclear if a shorter application time such as 15 s provides more benefits or risks.

## Conclusion

In summary, a hand gel based on 85% ethanol demonstrated a comprehensive bactericidal activity within 15 s. Future research will hopefully help to find out if a shorter application time for the post contamination treatment of hands provides more benefits or more risks.

## Competing interests

The first author is paid employee of Bode Chemie GmbH & Co. KG, Hamburg, Germany.

## Authors' contributions

GK designed the study and drafted the manuscript. AH acquired and analyzed the data, participated in design and coordination of the study, and helped to draft the manuscript. All authors read and approved the final manuscript.
